# Effects of practicing yoga on alexisomia: an open-label trial

**DOI:** 10.1186/s13030-022-00243-4

**Published:** 2022-06-03

**Authors:** Takakazu Oka, Battuvshin Lkhagvasuren

**Affiliations:** 1 Department of Psychosomatic Medicine, International University of Health and Welfare Narita Hospital, 852 Hatakeda, Narita, Chiba 286-8520 Japan; 2 Department of Psychosomatic Medicine, Graduate School of Medical Sciences, Kyushu University, Fukuoka, Japan

**Keywords:** Alexisomia, Alexithymia, Yoga, Shitsu-taikan-sho, STSS, TAS-20

## Abstract

**Background:**

Alexisomia refers to difficulties in the awareness and expression of somatic feelings. This idea was proposed by Dr. Yujiro Ikemi as a characteristic observed in patients with psychosomatic diseases and is based on his observations that patients with psychosomatic diseases have difficulty in the awareness and expression of not only their emotions, i.e., alexithymia, but also somatic feelings and sensations, i.e., alexisomia. He also proposed that treating alexisomia is important in the treatment of psychosomatic diseases and that yoga might help improve alexisomia. However, no study has investigated if yoga actually affects alexisomia. This open-label pilot study investigated whether practicing yoga in a class results in change in patients with alexisomia and alexithymia.

**Methods:**

The Shitsu-taikan-sho Scale (STSS) and the Toronto Alexithymia Scale (TAS-20) were administered to 305 participants, including 64 healthy participants, 111 participants who had subjective symptoms without abnormal findings, and 130 participants with chronic diseases. Participants were tested before and 3 months after attending yoga classes.

**Results:**

Yoga practice reduced the STSS and the TAS-20 difficulty in identifying feelings (DIF) subscale scores. Multiple linear regression indicated that a reduction in the TAS-20 DIF subscale scores predicted a decrease in the STSS score, whereas reductions in the STSS difficulty in identifying bodily feelings (DIB) and the lack of health management based on bodily feelings (LHM) subscale scores predicted a decrease in the TAS-20 scores.

**Conclusion:**

We found that regular yoga practice improves alexisomia. Yoga-induced improvement of alexisomia may be mediated, at least in part, by an improvement of DIF in alexithymia. Yoga would be a promising therapeutic approach to improve alexisomia.

**Supplementary Information:**

The online version contains supplementary material available at 10.1186/s13030-022-00243-4.

## Background

Alexisomia refers to difficulties in the awareness and expression of somatic feelings (for a review, see [1]). This concept was first proposed by Ikemi Y. as a characteristic observed in patients with psychosomatic diseases [2]. In 1973, P.E. Sifneos published the concept of alexithymia (difficulty in identifying and describing one’s emotions) as a characteristic of patients with psychosomatic diseases [3]. Soon after, Ikemi noted, “In many cases of alexithymia, where there is an observed difficulty in the awareness and expression of feelings, there also seems to be a difficulty in the awareness and expression of bodily feelings”. He coined the term alexisomia to designate the condition wherein people have difficulty in expressing how their bodies feel [4].

Ikemi observed characteristics of both alexithymia and alexisomia in patients with psychosomatic diseases, but he placed more importance on alexisomia. The concept of alexisomia emphasizes bodily (interoceptive/proprioceptive) awareness, whereas alexithymia emphasizes emotions in general. Ikemi hypothesized that body-oriented, somatopsychic approaches such as yoga, which alter proprioceptive afferent discharge, may be effective for alexithymic conditions and their related psychosomatic diseases [5]. He postulated the importance of integrating somatopsychic approaches and psychosomatic approaches, such as psychotherapy, in treating psychosomatic diseases [4, 5]. However, other than some case reports, no experimental studies have investigated whether body-oriented intervention actually improves the alexisomia of patients with psychosomatic diseases [6].

Yoga is a mind/body exercise that includes asana (poses), pranayama (breathing exercises), and meditation through which practitioners focus their attention on bodily feelings (slow movements, breathing, or relaxed/stretched muscle tension) and associated psychological feelings (calmness and peacefulness) [7]. We hypothesized that this mind/body approach of yoga ameliorates alexisomia. The STSS, a self-rating questionnaire, is a screening test that evaluates alexisomia without the need for a professional diagnosis [8, 9]. Therefore, to test our hypothesis in a pilot study, we investigated changes in the STSS scores of adult participants who began practicing yoga in yoga studios. Considering the close relation between alexisomia and alexithymia, we also investigated whether yoga practice affected alexithymia and whether improvement in alexisomia was associated with alexithymia and vice versa.

## Methods

### Study design

This study enrolled 390 adults who had not previously practiced yoga and began practicing it for the first time in yoga studios. They attended weekly yoga classes for three months. Participants completed two self-rating questionnaires, the STSS and the TAS-20, before the beginning of the first class and again after 3 months of attendance.

Participants were divided into three groups: healthy control participants (HC group; no subjective symptoms), participants with poor health (PH group; several subjective symptoms but having normal findings in physical examinations and medical tests), and participants with chronic disease (CD group; diagnosed with chronic diseases and on medication). Participants in the PH group did not receive any medical treatment, whereas those in the CD group received treatment for hypertension, low back pain, autonomic dysfunction, depression, and climacteric disorder, among others.

### Yoga classes

Yoga classes were held weekly, with a duration of 60 to 90 min. Most participants practiced yoga in class once a week and some also practiced at home. The classes included 5 to 20 participants and were taught by certified yoga therapists of the Japan Yoga Therapy Society according to the society’s guidelines. Each class had a similar structure, i.e., the yoga class included asanas (yoga poses), pranayamas (breathing exercises), meditation, isometric yoga poses (poses practiced with low impact isometric load and with long breathing [10, 11] that induce post-isometric relaxation [12]), and savasana (corpse pose). While practicing yoga, participants were encouraged to move slowly with breathing and to observe bodily sensations and feelings, which changed from tension to relaxation, comfort, calmness or peacefulness.

### Questionnaires

The STSS is a 23-item self-report questionnaire that assesses alexisomia and has three subscales: 1) difficulty in identifying bodily feelings (DIB), 2) overadaptation (OA), and 3) lack of health management based on bodily feelings (LHM). The STSS was developed in Japan and shows strong reliability and validity for assessing alexisomia [8]. Each item was evaluated on a 5-point Likert scale, ranging from 1 “definitely false” to 5 “definitely true”. The DIB subscale evaluates the failure to notice bodily sensations that act as warning signals during adaptation to external environments and that are necessary to maintain one’s homeostasis, as well as the failure to notice relaxed feelings that are necessary to prevent allostatic load. The OA subscale assesses the tendency to ignore warning signals from the body that result from prioritizing social demands and adapting to external environments despite feelings of fatigue, sleepiness, or a desire to rest. The LHM subscale evaluates habits related to maintaining daily health and assessing bodily sensations coming from physical conditions of the relaxation response [1, 8].

The TAS-20 is a 20-item self-report questionnaire with three subscales that evaluates alexithymia: 1) difficulty in identifying feelings (DIF), 2) difficulty in describing feelings (DDF), and 3) externally-oriented thinking (EOT) [13]. The Japanese version of the TAS-20 has been shown to be reliable and valid for use with general and clinical populations [14].

The STSS total and subscale scores have strong correlations with the TAS-20 total and subscale scores, especially the STSS DIB subscale and the TAS-20 DIF subscale scores [8].

### Statistical analysis

Results are presented as mean ± standard deviation (SD). The non-parametric paired samples Wilcoxon signed-rank test was used to assess the effects of yoga on alexisomia and alexithymia in comparison of the STSS and TAS-20 scores before and after 3 months of regular yoga practice. Two-way repeated ANOVA was used to examine differences in changes in the STSS and TAS-20 scores before and after the yoga practice, considering time effects and interactions between the groups. Correlations between continuous variables were assessed by Pearson’s correlation. Multiple linear regression with the enter method was conducted to examine whether age, sex, and changes in the TAS-20 subscale scores (independent variables) predicted changes in the STSS scores (dependent variables). To minimize type 1 errors across questionnaire scores, p values were adjusted for multiple comparisons by the use of an adaptive linear step-up procedure that controls the false discovery rate [15]. Effect sizes for significant differences in the values were calculated using the Wilcoxon *r*, Cramer’s *V*, and eta squared (*η*^*2*^) with a 95% confidence interval (CI) as appropriate. Statistical significance was set at *p* < 0.05, and all tests were two-tailed. Data were analyzed using SPSS v.21.0.

## Results

### Demographics

Three hundred ninety adults who had begun practicing yoga for the first time were enrolled. Among these participants, 57 discontinued the class within three months and 28 were excluded from the analysis due to missing values, leaving the data of 305 participants available for the final analysis. The participants were aged from 21 to 92 years: 64 in the H group, 111 in the PH group, and 130 in the CD group (Table 1). The male/female ratio was not different among the groups. However, there was a significant difference in age among the three groups (*p* < 0.001, F = 16.35).

**Table 1:** Effects of yoga on the STSS and TAS-20 scores

Group		Total				H				PH				CD			
		Values	UPV	FPV	ES	Values	UPV	FPV	ES	Values	UPV	FPV	ES	Values	UPV	FPV	ES
**Number** **Sex,** m / f		305 30 / 275	0.064		0.13	64 11 / 53				111 7 / 104				130 12 / 118			
**Age,** years		53.7 ± 15.0	< 0.001		0.10	53.6 ± 14.4				48.0 ± 14.6				58.6 ± 14.1			
**STSS, Total**	Pre	59.2 ± 11.2	< 0.001	< 0.001	0.26	57.2 ± 10.9	0.032	0.043	0.12	59.4 ± 10.8	< 0.001	< 0.001	0.24	60.1 ± 11.6	0.001	0.003	0.19
	Post	56.2 ± 11.1				54.8 ± 10.2				55.9 ± 11.2				57.2 ± 11.3			
**DIB**	Pre	22.9 ± 5.9	0.001	0.002	0.13	22.0 ± 6.2	0.027	0.042	0.28	22.4 ± 5.9	0.033	0.043	0.20	23.7 ± 5.7	0.095	0.117	0.15
	Post	21.9 ± 5.9				20.7 ± 5.7				21.5 ± 6.1				23.0 ± 5.8			
**OA**	Pre	17.4 ± 5.2	0.003	0.006	0.17	17.1 ± 4.6	0.661	0.661	0.03	17.7 ± 4.8	0.001	0.003	0.18	17.2 ± 5.8	0.214	0.228	0.07
	Post	16.7 ± 5.6				16.8 ± 4.6				16.3 ± 5.1				17.0 ± 6.1			
**LHM**	Pre	18.9 ± 4.5	< 0.001	< 0.001	0.27	18.0 ± 4.1	0.159	0.181	0.08	19.3 ± 4.5	< 0.001	< 0.001	0.21	19.1 ± 4.8	< 0.001	< 0.001	0.22
	Post	17.8 ± 4.8				17.9 ± 6.9				18.1 ± 4.3				17.4 ± 3.9			
**TAS-20, Total**	Pre	50.5 ± 9.5	0.596	0.810	0.03	49.3 ± 8.1	0.700	0.810	0.02	50.2 ± 10.0	0.708	0.810	0.02	51.4 ± 9.7	0.382	0.744	0.05
	Post	49.9 ± 8.2				48.8 ± 7.9				50.1 ± 8.2				50.4 ± 8.2			
**DIF**	Pre	16.5 ± 5.2	0.026	0.422	0.05	15.2 ± 4.0	0.421	0.744	0.05	16.7 ± 5.0	0.325	0.743	0.06	17.0 ± 5.7	0.057	0.427	0.11
	Post	15.9 ± 4.8				14.8 ± 4.1				16.4 ± 5.2				16.1 ± 4.7			
**DDF**	Pre	14.4 ± 3.4	0.230	0.612	0.04	13.9 ± 3.0	0.848	0.848	0.01	14.4 ± 3.0	0.465	0.744	0.04	14.7 ± 3.8	0.217	0.612	0.07
	Post	14.1 ± 3.2				14.0 ± 3.2				14.0 ± 3.3				14.3 ± 3.2			
**EOT**	Pre	19.6 ± 4.0	0.215	0.612	0.06	20.2 ± 3.7	0.828	0.848	0.01	19.6 ± 4.7	0.080	0.427	0.10	19.7 ± 3.5	0.678	0.810	0.02
	Post	19.8 ± 3.7				20.0 ± 3.4				19.6 ± 4.2				19.9 ± 3.4			

*CD* Chronic disease, *DDF* Difficulty in describing feelings, *DIB* Difficulty in identifying bodily feelings, *DIF* Difficulty in identifying feelings, *EOT* Externally-oriented thinking, *ES* Effect size between Pre and Post (Wilcoxon *r/* Cramer’s *V*/ Eta squared *η*.^*2*^), *f* Female, *FPV* False discovery rate–adjusted *p* values for multiple comparisons (Pre vs Post, Wilcoxon signed rank test), *H* Healthy, *LHM* Lack of health management based on bodily feelings, *m* Male, *OA* Overadaptation, *PH* Poor health, *Post* After practicing yoga for 3 months, *Pre* Before starting to practice yoga, *STSS* The Shitsu-taikan-sho Scale, *TAS-20* The 20-item Toronto Alexithymia Scale, *UPV* Unadjusted *p* values

The number of dropouts in each group was 13 (15.5%) in the H group, 23 (16.3%) in the PH group, and 21 (12.7%) in the CD group. Rates of dropouts were not different among the groups (*p* = 0.718, *χ*^*2*^ = 0.663).

#### STSS

The mean STSS total score in the study population was decreased from 59.2 points to 56.2 points through yoga practice. Table 1 shows the Wilcoxon signed-rank test results indicating that the STSS total and subscale scores (DIB, OA, and LHM) decreased during the period of yoga practice among all participants (*p* < 0.001, *p* = 0.001, *p* = 0.003, and *p* < 0.001, respectively). In all groups (H, PH, and CD), the STSS total score was reduced after yoga practice (*p* = 0.032, *p* < 0.001, and *p* = 0.001, respectively). The PH group showed improvement in all subscales, whereas the H and CD groups improved on the DIB and LHM subscales, respectively. The STSS scores of the PH and CD groups were higher than those of the H group (Table 1).

**Fig. 1:**
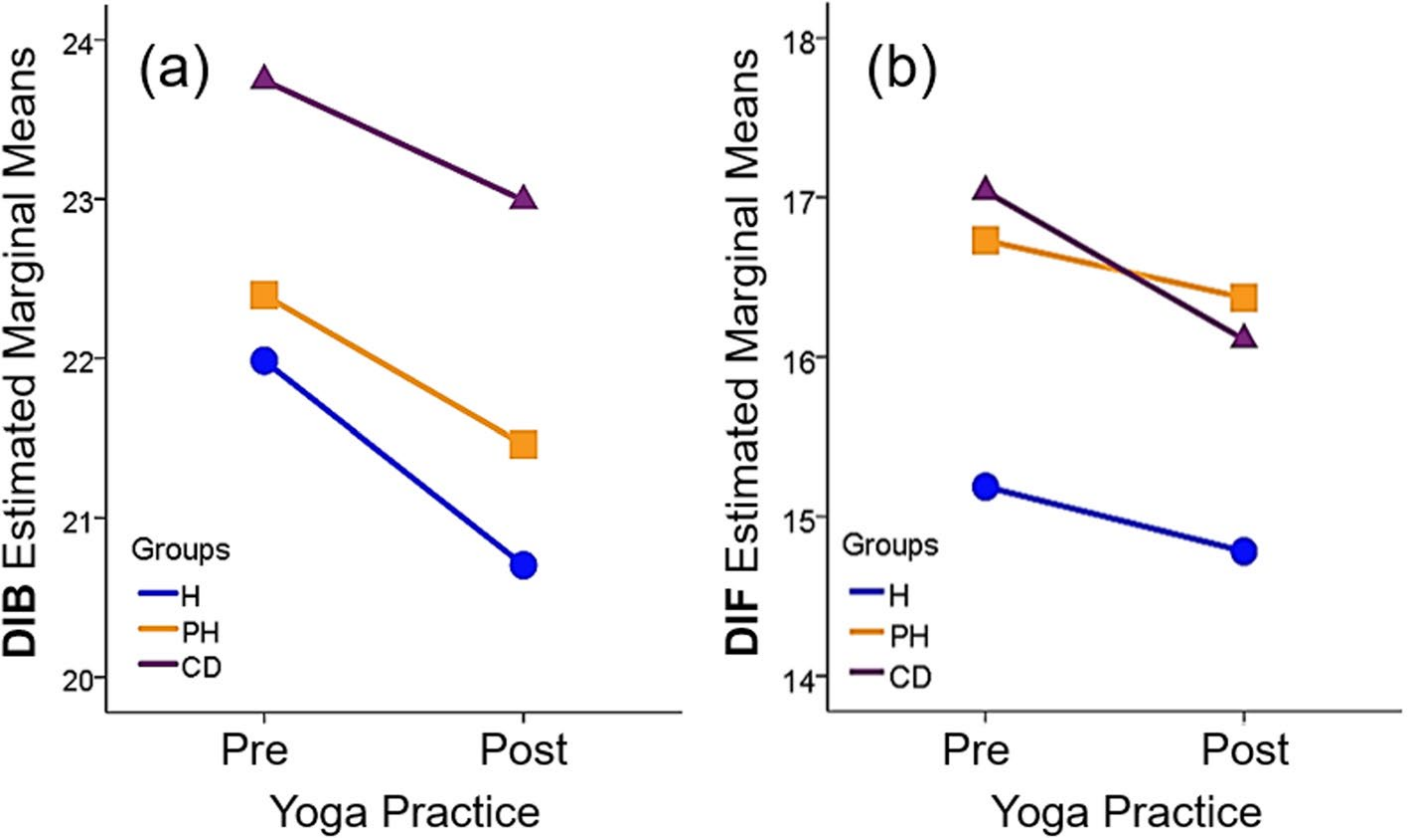
Effect of yoga on the STSS, DIB subscale score (**a**) and the TAS-20, DIF score (**b**). **a**: Changes in the estimated marginal means of DIB before and after yoga practice; **b**: Changes in the estimated marginal means of DIF before and after yoga practice; CD: chronic disease; H: healthy; PH: poor health; Post: after practicing yoga for 3 months; Pre: before starting to practice yoga

In Table 2, further analysis using a two-way repeated ANOVA confirmed that the yoga practice reduced the STSS total and subscale scores during the period of yoga practice (within-subject effects: *p* < 0.001, *p* = 0.001, *p* = 0.016, and *p* < 0.001, respectively). The change in DIB over time (ΔDIB) was significantly different between the groups (*p* = 0.023, F = 3.804), and there was no significant interaction effect. These results suggest that yoga improves alexisomia. Furthermore, Tukey’s post-hoc test indicated that the difference between the estimated marginal mean of the H group (21.3 ± 0.7) and that of the CD group (23.4 ± 0.5) was significant (*p* = 0.037) but there was no difference between the PH group (21.9 ± 0.5) and the H and CD groups. These results suggest that the H group benefited in particular from yoga practice for DIB (Fig. 1a).

**Table 2:** Effects of yoga practice on the STSS and TAS-20 of the H, PH, and CD groups assessed by two-way repeated ANOVA

Scale		Overall *p* value	F value	Within-subject	Inter-action	ES (*η*^*2*^)	Power
**STSS**	**Total**	0.229	1.480	< 0.001	0.744	0.010	0.32
	**DIB**	0.023	3.804	0.001	0.776	0.025	0.69
	**OA**	0.973	0.027	0.016	0.137	0.001	0.05
	**LHM**	0.468	0.761	< 0.001	0.094	0.005	0.18
**TAS-20**	**Total**	0.298	1.214	0.246	0.589	0.008	0.26
	**DIF**	0.045	3.125	0.026	0.528	0.020	0.60
	**DDF**	0.367	1.006	0.276	0.595	0.007	0.22
	**EOT**	0.320	1.143	0.389	0.842	0.008	0.25

*DDF* Difficulty in describing feelings, *DIB* Difficulty in identifying bodily feelings, *DIF* Difficulty in identifying feelings, *EOT* Externally-oriented thinking, *ES* Effect size between Pre and Post (Eta squared *η*^*2*^), *LHM* Lack of health management based on bodily feelings, *OA* Overadaptation, *STSS* The Shitsu-taikan-sho Scale, *TAS-20* The 20-item Toronto Alexithymia Scale

#### TAS-20

In contrast, there was no difference between the TAS-20 total and subscale scores before and after yoga practice in any group or for all participants, by the non-parametric paired test (Table 1). However, two-way repeated ANOVA revealed that the DIF score decreased in all participants (within-subject effect: *p* = 0.026). Furthermore, the change in DIF across time (ΔDIF) was different between the groups (*p* = 0.045, F_5_ = 3.12), and there was no interaction effect (Table 2). These findings suggest that yoga improves critical components of alexithymia, i.e., difficulty in identifying feelings. Furthermore, Tukey’s post-hoc test suggests that the estimated marginal mean of the H group (15.0 ± 0.6) differed significantly from those of the PH (16.6 ± 0.4) and CD groups (16.6 ± 0.4) (*p* = 0.049 and *p* = 0.038, respectively), but there was no difference between the PH and CD groups (*p* = 0.999). We also found that the mean DIF before and after yoga differed between the groups in one-way ANOVA (*p* = 0.022 and *p* = 0.027, respectively).

### Factors to STSS

Table 3 shows the results of multiple linear regression with the enter method indicating that the ΔDIF score of TAS-20 was a predictive factor. It accounted for 14% of the variance of the ΔSTSS total score (F_5_ = 9.9, *p* < 0.001). This model showed that there was no multicollinearity detected between the tested variables (1 < VIF < 2.5; tolerance < 10); no outliers (Cook’s distance < 1; standard residuals <  ± 3.3); and the independence assumption was satisfied (1.5 < Durbin-Watson < 2.5). The distribution of the residuals satisfied normality assumptions. The variance of the model was constant and homoscedasticity was not violated. Similarly, the ΔDIF subscale score of the TAS-20 predicted the ΔSTSS subscale scores, including the ΔDIB, ΔOA and ΔLHM scores (Table 3). No other subscale scores of TAS-20 were associated with the ΔSTSS scores. These results suggest that a reduced difficulty in identifying feelings contributed to an improvement in alexisomia. Furthermore, all ΔSTSS total and subscale scores were correlated with the ΔDIF subscale score before versus after the yoga intervention (Table 3). This indicates that alexisomia and its subscales are correlated with alexithymia, especially for the subscale of difficulty in identifying feelings. Moreover, we analyzed the contribution of the TAS-20 subscale scores before and after yoga practice to the changes in the STSS total and subscale scores (Appendix 1). The results suggest that only DIF before yoga predicts the ΔSTSS total and ΔDIB, confirming the above-mentioned findings.

**Table 3:** Multiple linear regression for the ΔSTSS scores of all subjects. *N* = 305 (independent variables: age, sex, ΔDIF, ΔDDF, and ΔEOT)

Independent variables	Dependent variables (ΔSTSS)			
	**ΔTotal**	**ΔDIB**	**ΔOA**	**ΔLHM**
Mean ± SD	3.03 ± 9.09	0.93 ± 4.83	0.69 ± 4.57	1.16 ± 4.62
***Model summary**** (*r^2^*)*	0.142	0.121	0.043	0.036
F value	9.860	8.235	2.662	2.241
Durbin-Watson	1.923	2.097	1.850	1.946
Cook’s distance	0.107	0.076	0.90	0.128
*p* value	< 0.001	< 0.001	0.023	0.05
Constant	5.521	1.504	1.600	1.734
**Age**	(mean ± SD: 53.69 ± 15.05; VIF: 1.020)			
r	-0.101	0.003	-0.083	-0.071
B	-0.055	0.004	-0.023	-0.022
*p* value	0.093	0.820	0.195	0.219
**Sex**	(male/female: 30/275; VIF: 1.010)			
r	0.011	-0.032	0.014	0.023
B	-0.002	-0.549	0.085	0.270
*p* value	0.999	0.535	0.922	0.760
**ΔDIF**	(mean ± SD: 0.61 ± 4.23; VIF: 1.379)			
r	0.364^***^	0.342^***^	0.189^**^	0.161^**^
B	0.731	0.368	0.198	0.171
*p* value	< 0.001	< 0.001	0.006	0.019
**ΔDDF**	(mean ± SD: 0.26 ± 3.14; VIF: 1.361)			
r	0.213^***^	0.211^***^	0.105	0.070
B	0.110	0.077	0.021	-0.028
*p* value	0.545	0.428	0.827	0.773
**ΔEOT**	(mean ± SD: -0.23 ± 3.52; VIF: 1.035)			
r	0.058	0.027	-0.003	0.091
B	0.025	-0.40	-0.034	0.099
*p* value	0.859	0.595	0.652	0.193
**ΔTAS-20 total** r	0. 311^***^	0. 285^***^	0. 157^**^	0. 146^*^

Δ: differences between scores before and after 3 months of yoga practice; *p < 0.05; **p < 0.01; ***p < 0.001 for correlation coefficients (r) with the Pearson’s correlation, *DDF* Difficulty in describing feelings, *DIB* Difficulty in identifying bodily feelings, *DIF* Difficulty in identifying feelings, *EOT* Externally-oriented thinking, *LHM* Lack of health management based on bodily feelings, *OA* Overadaptation, *p value* Multiple linear regression with the enter method, *SD* Standard deviation, *STSS* The Shitsu-taikan-sho Scale, *TAS-20* The 20-item Toronto Alexithymia Scale, *VIF* Variance inflation factor

### Factors to TAS-20

Table 4 shows the results of multiple linear regression with the enter method. They indicate that the ΔDIB and ΔLHM scores of STSS were predictive factors, and they accounted for 10% of the variance of the ΔTAS-20 total score (*p* < 0.001, F_5_ = 6.64). This model showed there was no multicollinearity between the tested variables (1 < VIF < 2.5; tolerance < 10), there were no outliers (Cook’s distance < 1; standard residuals <  ± 3.3), and the independence assumption was satisfied (1.5 < Durbin-Watson < 2.5). The distribution of the residuals satisfied normality assumptions. The variance of the model was constant and homoscedasticity was not violated. Similarly, the ΔDIB and ΔLHM scores of the ΔSTSS predicted the ΔDIF subscale score of TAS-20 (Table 4). Moreover, ΔDIB alone predicted the ΔDDF subscale score of TAS-20. In contrast, ΔOA of STSS was not associated with any ΔTAS-20 score, although it correlated with ΔDIF. ΔEOT had no correlation with any ΔSTSS score (Table 4). We also analyzed the contribution of the STSS subscale scores before and after yoga practice to the changes in the TAS-20 total and subscale scores (Appendix 2). The results suggest that LHM before yoga predicts all of the ΔTAS-20 scores, whereas DIB before yoga predicts only ΔDIF. These results suggest that reduced alexisomia contributed to an improvement in the alexithymia components, including difficulty in identifying and describing feelings.

**Table 4:** Multiple linear regression for the ΔTAS-20 scores of all subjects. *N*=305 (independent variables: age, sex, ΔDIB, ΔOA, and ΔLHM)

Independent variables	Dependent variables (ΔTAS-20)			
	**ΔTotal**	**ΔDIF**	**ΔDDF**	**ΔEOT**
Mean ± SD	0.58 ± 7.72	0.61 ± 4.23	0.26 ± 3.14	-0.23 ± 3.52
***Model summary*** (r^2^)	0.100	0.139	0.049	0.018
F value	6.640	9.639	3.111	1.094
Durbin-Watson	2.092	2.096	2.087	2.033
Cook’s distance	0.060	0.078	0.090	0.136
*p* value	< 0.001	< 0.001	0.009	0.364
Constant	-1.991	0.261	-0.838	-1.550
**Age**	(mean ± SD: 53.69 ± 15.05; VIF: 1.020)			
r	0.024	-0.033	0.007	0.090
B	-0.055	-0.005	-0.023	0.022
*p* value	0.093	0.724	0.195	0.101
**Sex**	(male/female: 30/275; VIF: 1.010)			
r	0.009	0.004	0.031	-0.006
B	0.449	0.113	0.085	0.014
*p* value	0.753	0.883	0.922	0.984
**ΔDIB**	(mean ± SD: 0.93 ± 4.83; VIF: 1.158)			
r	0.285^***^	0.342^***^	0.211^**^	0.027
B	0.407	0.270	0.128	0.017
*p* value	< 0.001	< 0.001	0.001	0.710
**ΔOA**	(mean ± SD: 0.69 ± 4.57; VIF: 1.166)			
r	0.157^**^	0.189^***^	0.105	-0.003
B	0.094	0.059	0.021	-0.010
*p* value	0.350	0.272	0.622	0.838
**ΔLHM**	(mean ± SD: 1.16 ± 4.62; VIF: 1.017)			
r	0.146^**^	0.161^**^	0.070	0.091
B	0.019	0.116	0.034	0.074
*p* value	0.030	0.020	0.379	0.095
**ΔSTSS total** r	0. 311^***^	0. 364^***^	0. 213^**^	0. 058

Δ: differences between scores before and after 3 months of yoga practice; *p < 0.05; **p < 0.01; ***p < 0.001 for correlation coefficients (r) with the Pearson’s correlation, *DDF* Difficulty in describing feelings, *DIB* Difficulty in identifying bodily feelings, *DIF* Difficulty in identifying feelings, *EOT* Externally-oriented thinking, *LHM* Lack of health management based on bodily feelings, *OA* Overadaptation, *p value* Multiple linear regression with the enter method, *SD* Standard deviation, *STSS* The Shitsu-taikan-sho Scale, *TAS-20* The 20-item Toronto Alexithymia Scale, *VIF* Variance inflation factor

## Discussion

The present study is the first to investigate the effect of yoga on alexisomia. We demonstrated that practicing yoga for 3 months decreased the STSS total scores of all of our study groups. Because the STSS scores in the PH and CD groups were higher than that of the H group, which was identical to that of healthy university students (56 ± 10) in our previous study [8], the findings suggest that yoga can reduce the degree of alexisomia of those with poor health or a chronic disease to a healthy level.

The mechanisms underlying the improvement of alexisomia are still unclear. One possibility may related to how yoga is instructed. During class, instructors encourage participants to be mindful of inner bodily sensations, such as breathing and muscle tension/relaxation. It is reasonable to hypothesize that these practices enhance interoceptive awareness, which leads to improved alexisomia, especially the DIB component. Studies on whether yoga can improve interoceptive awareness are limited [16, 17], and future studies are necessary to investigate this hypothesis.

Yoga also improved the OA and LHM subscale scores. These changes may also be linked to the way yoga is practiced. The OA subscale assesses the tendency to ignore warning signals from the body that result from prioritizing social demands, despite fatigue or the desire for rest. The LHM subscale assesses habits related to daily health maintenance. After practicing many poses, participants observe and “taste” inner feelings, which are usually relaxed, comfortable, calm, and peaceful ones, during meditation and savasana. These yoga practices may gradually shift participants’ mindset, coping, and lifestyle from meeting social demands and ignoring physical needs to following the “voice of the body”.

In addition to the STSS scores, yoga also decreased the DIF subscale score of the TAS-20 according to the results of two-way repeated ANOVA. Several studies have investigated yoga’s effects on alexithymia [18, 19], but their findings are controversial. One study showed that yoga reduced the TAS-20 score of patients with mild to moderate depression and anxiety [19], whereas another study demonstrated that yoga did not change the TAS-20 score of those with myalgic encephalomyelitis/chronic fatigue syndrome [18]. These differences may be due to different study populations or degrees of alexithymia. Considering that the TAS-20 score of a healthy Japanese sample was 48 ± 9 [14], participants in this study exhibited a lower level of alexithymia compared to healthy people. This might explain why yoga had limited effects on the TAS-20 total and subscale scores in this study. To determine if yoga can affect alexithymia, future studies of people with alexithymia will be necessary.

In this study, we found that the ΔSTSS scores were positively correlated with the ΔTAS-20 scores, particularly with ΔDIF. These results support our previous study results in which we evaluated the external validity of STSS using TAS-20 and found correlations between the measures [8]. The present study used multiple linear regression to further analyze these associations and found that 14% of the variance of the ΔSTSS total score was predicted by ΔDIF. Furthermore, ΔDIF predicted changes in all STSS subscales, i.e., DIB, OA, and LHM. In turn, regression analysis for alexithymia suggests that the improvement of alexithymia (DIF and DDF) of TAS-20 due to yoga can partially explain the improvement of alexisomia (DIB and LHM). These results indicate that STSS is distinct from TAS-20 structurally, but also that it is partially convergent. During yoga, participants are directed to focus on inner feelings, and this focus is associated with attention to changes in proprioception/interoception and enhanced emotional awareness. These practices may help improve alexithymia, especially the difficulty in identifying feelings component and may contribute to improving alexisomia.

There are several limitations in this study. First, the data collected were self-reported measures. It is controversial whether self-administered questionnaires such as the TAS-20 and STSS can be used as screening tools for alexithymia or alexisomia [1, 14, 20–22]. Therefore, although this study suggested that yoga improved alexisomia, this finding needs to be confirmed through medical interviews by experts in psychosomatic medicine who are familiar with this concept. Second, this is an open-label trial. Therefore, to determine whether yoga definitely provides these changes, it will be necessary to compare the changes in these values with a control group in a randomized, controlled trial. Because participants of the CD group were on medication, there remains a possibility that their medical treatment had some effect on the STSS scores. Third, because we measured only STSS and TAS-20 in this study, it is difficult to explain how yoga affected alexisomia and alexithymia differently. To address this question and discriminate the effects of yoga on the specific characteristics of alexisomia and alexithymia, future studies are needed that include more general physical and mental health questionnaires. Fourth, because yoga has many styles and yoga studios have independent programming, generalizing our conclusions to all types of yoga is difficult. Fifth, our participants attended yoga classes of their own volition, meaning they were motivated. Whether yoga would have the same effects on those without a prior interest in yoga is unclear.

Despite these limitations, we believe that this is the first study to show that yoga can reduce STSS scores.

## Conclusion

This study suggests that yoga is promising for improving the alexisomia of adults.

## Supplementary Information

**Additional file 1.**

**Additional file 2.**

## Data Availability

Data sharing is not applicable.

## Abbreviations

STSS: The Shitsu-taikan-sho Scale
TAS-20: Toronto Alexithymia Scale-20
DIF: Difficulty in identifying feelings
HC: Healthy control
PH: Participants with poor health
CD: Participants with chronic disease
DIB: Difficulty in identifying bodily feelings
OA: Overadaptation
LHM: Lack of health management based on bodily feelings
DDF: Difficulty in describing feelings
EOT: Externally-oriented thinking
