# Supplementary material for: Effects of practicing yoga on alexisomia: an open-label trial
Source: Biopsychosoc Med. 2022 Jun 3;16:14. doi: 10.1186/s13030-022-00243-4 (PMC9166595; doi:10.1186/s13030-022-00243-4)
Supplement: Supplementary file 2 — Additional file 2. [file 13030_2022_243_MOESM2_ESM.docx]

**Appendix 2** Multiple linear regression for the ΔTAS-20 total and subscale scores (n=305)

| **Regression variables** | | **Dependent variables (ΔTAS-20)** | | | | | | | | | | | |
| --- | --- | --- | --- | --- | --- | --- | --- | --- | --- | --- | --- | --- | --- |
|  |  | **ΔTotal** | | | **ΔDIF** | | | **ΔDDF** | | | **ΔEOT** | | |
|  |  | **r^2^** | **p value** | **predictors** | **r^2^** | **p value** | **predictors** | **r^2^** | **p value** | **predictors** | **r^2^** | **p value** | **predictors** |
| **Independent variables** | **Pre STSS** | 0.04 | 0.035 | Pre LHM (p=0.004) | 0.04 | 0.048 | Pre DIB (p=0.031) Pre LHM (p=0.019) | 0.02 | 0.207 | Pre LHM (p=0.037) | 0.01 | 0.127 | Pre LHM (p=0.039) age (p=0.022) |
|  | **Post STSS** | 0.02 | 0.358 | NA | 0.03 | 0.141 | NA | 0.01 | 0.616 | NA | 0.02 | 0.443 | NA |
|  | **ΔSTSS** | 0.1 | <0.001 | ΔDIB (p<0.001) ΔLHM (p=0.03) | 0.14 | <0.001 | ΔDIB (p<0.001) ΔLHM (p=0.02) | 0.05 | 0.009 | ΔDIB (p=0.001) | 0.02 | 0.364 | NA |

*Δ* differences between scores before and after yoga practice, *DDF* difficulty in describing feelings, *DIB* difficulty in identifying bodily feelings, *DIF* difficulty in identifying feelings, *EOT* externally-oriented thinking, *LHM* lack of health management based on bodily feelings, *NA* not available, *OA* overadaptation, *p value* multiple linear regression with the enter method, *Pre* scores before yoga practice, *Post* scores after yoga practice, *r^2^* R-squared or a goodness of fit, *STSS* the Shitsu-taikan-sho Scale, *TAS-20* the 20-item Toronto Alexithymia Scale
